# Induction of potent systemic anti-melanoma immunity through intratumoral CD40 activation and checkpoint blockade

**DOI:** 10.1186/2051-1426-3-S2-P313

**Published:** 2015-11-04

**Authors:** Manisha Singh, Zhimin Dai, Hiep Khong, Christina Vianden, Mark Cantwell, Willem Overwijk

**Affiliations:** 1 University of Texas - MD Anderson Cancer Center, Houston, TX, USA; 2 Memgen, LLC, San Diego, CA, USA

## Purpose

Agonistic CD40 antibodies generate strong tumor specific CD8 T cell response and anti-tumor activity; however systemic anti-CD40 therapy has been associated with cytokine release syndrome and liver toxicity. We studied the anti-melanoma activity and mechanism of action of a recombinant adenovirus expressing a stabilized version of CD40L (rAd.CD40L) by local intratumoral delivery approach to treat metastatic melanoma.

## Experimental design

Mice bearing established B16 melanomas were treated intratumorally with rAd.CD40L (ISF35) or rAd5 control virus and received anti-PD1 plus anti-CTLA-4 systemically. Anti-tumor effects of mono or combination therapies were determined by mice survival and tumor growth measurement. The mechanistic contribution of immune cells to this therapy was determined by using antibody blockades. Immune cell infiltrates in tumor and expression of negative regulators on these cells were analyzed by flow cytometry.

## Results

Intratumoral administration of rAd.CD40L generated systemic anti-tumor immunity mediated by CD8 T cells and suppressed both injected and distant uninjected wild-type B16.F10 melanomas. However, tumors did not completely regress after therapy. Analysis of tumor-infiltrating leukocytes revealed that almost 100% of tumor-infiltrating CD8 T cells in the rAd.CD40L-treated group had up-regulation of the T cell inhibitory molecule PD-1. Combined treatment with rAd.CD40L plus anti-PD1 was highly synergistic and induced higher number of melanoma specific CD8 T cells systemically. Concomitant CTLA-4 blockade further improved the efficacy of treatment and led to complete regression of melanoma in about 50% of mice and generated memory CD8 T cells response.

## Conclusion

Immunotherapy based on intratumoral CD40 activation is potentiated by PD-1 and CTLA-4 blockade and this combination generates functional and long-lasting anti-tumor CD8 T cell immunity that systemically suppresses melanoma metastases. These results suggest combination of rAd.CD40L with checkpoint blockade inhibitors may offer a promising immunotherapeutic option of metastatic melanoma that does not respond to checkpoint blockage monotherapy.

